# Vitamin C Deficiency Reduces Neurogenesis and Proliferation in the SVZ and Lateral Ventricle Extensions of the Young Guinea Pig Brain

**DOI:** 10.3390/antiox11102030

**Published:** 2022-10-14

**Authors:** Nery Jara, Manuel Cifuentes, Fernando Martínez, Iván González-Chavarría, Katterine Salazar, Lucas Ferrada, Francisco Nualart

**Affiliations:** 1 Laboratorio de Neurobiología y Células Madre, NeuroCellT, Departamento de Biología Celular, Facultad de Ciencias Biológicas, Universidad de Concepción, Concepción 4030000, Chile; 2 Department of Cell Biology, Genetics and Physiology, University of Malaga, 29010 Malaga, Spain; 3 Centro de Microscopía Avanzada CMA BIO BIO, Universidad de Concepción, Concepción 4030000, Chile; 4 Laboratorio de Lipoproteínas y Cáncer, Departamento de Fisiopatología, Facultad de Ciencias Biológicas, Universidad de Concepción, Concepción 4030000, Chile

**Keywords:** vitamin C, ascorbic acid, subventricular zone, lateral ventricle extensions, adult neurogenesis

## Abstract

Although scurvy, the severe form of vitamin C deficiency, has been almost eradicated, the prevalence of subclinical vitamin C deficiency is much higher than previously estimated and its impact on human health might not be fully understood. Vitamin C is an essential molecule, especially in the central nervous system where it performs numerous, varied and critical functions, including modulation of neurogenesis and neuronal differentiation. Although it was originally considered to occur only in the embryonic brain, it is now widely accepted that neurogenesis also takes place in the adult brain. The subventricular zone (SVZ) is the neurogenic niche where the largest number of new neurons are born; however, the effect of vitamin C deficiency on neurogenesis in this key region of the adult brain is unknown. Therefore, through BrdU labeling, immunohistochemistry, confocal microscopy and transmission electron microscopy, we analyzed the proliferation and cellular composition of the SVZ and the lateral ventricle (LVE) of adult guinea pigs exposed to a vitamin-C-deficient diet for 14 and 21 days. We found that neuroblasts in the SVZ and LVE were progressively and significantly decreased as the days under vitamin C deficiency elapsed. The neuroblasts in the SVZ and LVE decreased by about 50% in animals with 21 days of deficiency; this was correlated with a reduction in BrdU positive cells in the SVZ and LVE. In addition, the reduction in neuroblasts was not restricted to a particular rostro–caudal area, but was observed throughout the LVE. We also found that vitamin C deficiency altered cellular morphology at the ultrastructural level, especially the cellular and nuclear morphology of ependymal cells of the LVE. Therefore, vitamin C is essential for the maintenance of the SVZ cell populations required for normal activity of the SVZ neurogenic niche in the adult guinea pig brain. Based on our results from the guinea pig brain, we postulate that vitamin C deficiency could also affect neurogenesis in the human brain.

## 1. Introduction

Scurvy is a disease caused by severe vitamin C deficiency produced in humans and animal models that cannot produce endogenous vitamin C and must obtain it from the diet [1]. Although the incidence of scurvy has been declining for decades, the prevalence of subclinical vitamin C deficiency is much higher than previously estimated [2,3] and the impact on human health might not be fully appreciated. Vitamin C is an essential antioxidant molecule, especially in the central nervous system, where it performs numerous and varied functions [4]. For example, vitamin C, which is taken up through specialized membrane transporters [5], modulates neurogenesis (i.e., the genesis of new neurons or neuroblasts) [6] and neuronal differentiation (i.e., maturation of neuroblasts into fully functional neurons) [5,6].

The vitamin C transporter, SVCT2, is expressed in precursor cells of different embryonic and postnatal brain regions. During the beginning of the embryonic neurogenic period (E13), SVCT2 is expressed in the ventricular zone (VZ) and in the subventricular zone (SVZ), specifically in the radial glia cell body, and its high expression is maintained throughout the neurogenic period [7]. SVCT2 is also expressed in precursor cells during postnatal development of the cerebellum [8]. In postnatal cerebellum-derived neurospheres, the expression of nestin is correlated with SVCT2, demonstrating the transporter expression in cerebellar neural stem-like cells [8]. In addition, transient progenitors of adult mouse SVZ and RMS also express SVCT2, suggesting that vitamin C may be involved in a function along these neurogenic regions [9]. Treatment with vitamin C or its deficiency has also shown interesting findings. Initially, vitamin C treatment of embryonic stem cells and mesencephalic precursor cells was shown to increase the expression of genes involved in neurogenesis, differentiation and neurotransmission [10,11]. In vitro treatment of adult SVZ-derived neurospheres [9] and postnatal cerebellum-derived neurospheres [8] with vitamin C potentiates the neuronal phenotype by increasing expression of the neuronal markers. Moreover, the overexpression of SVCT2 in the mouse N2a cell line, through the incorporation of vitamin C and the subsequent phosphorylation of MAP-ERK1/2, induces the appearance of a differentiated phenotype, characterized by the development of filopodia and MAP-2-positive processes [12]. Furthermore, in the subgranular zone (SGZ), vitamin C deficiency reduces the number of neurons in the hippocampus of guinea pigs, altering spatial memory [13].

Although initially considered to occur only during infancy, it is now widely recognized that neurogenesis also takes place in the adult brain, mainly in two neurogenic niches: the SVZ in the lateral walls of the lateral ventricles (LV) [14,15] and the SGZ in the dentate gyrus of the hippocampus [16]. Of these regions, the SVZ stands out as the most extensive neurogenic area that harbors a greater number of neural stem cells (NSCs) [17]. The cellular architecture and composition of the SVZ has been described in several species. In mice, it consists mainly of four types of cells. The SVZ astrocytes or B cells correspond to NSCs, proliferating and originating transient progenitors or C cells. C cells, in turn, proliferate rapidly and differentiate into neuroblasts. Finally, ependymal cells correspond to a simple cuboidal epithelium that separates the ventricular cavity from the other cells of the SVZ [17,18]. 

Neuroblasts that originate from the SVZ are destined to form interneurons in the olfactory bulb (OB) [19]. To reach the OB, neuroblasts must migrate through a pathway known as the rostral migration stream (RMS) [19,20]. In humans, there is controversy regarding the existence of an RMS; however, neuroblasts migrating around a small ventricular cavity that connects the LV with the OB, named the lateral ventricle extension (EVL), have been described [21,22].

Numerous studies show that the SVCT2 transporter is expressed in progenitor cells and that vitamin C influences differentiation and neurogenesis [7,8,9,12]; however, the effect of this molecule or its deficiency on the adult neurogenesis that occurs in the SVZ as well as the effect on the cellular architecture and composition of this neurogenic region is unknown. Thus, in the present study, we describe the in vivo effect of vitamin C deficiency on adult neurogenesis in the SVZ, to later describe the in situ effects on the cellular architecture and composition of SVZ and EVL of the guinea pig brain. We use this model because the cellular architecture of SVZ and the presence of an EVL in the guinea pig brain is similar to that described in the human brain [23]. Additionally, guinea pigs do not synthesize vitamin C and must acquire it from their diet in a fashion similar to humans, which allows for modeling of vitamin C deficiency [1,24,25].

## 2. Materials and Methods

### 2.1. Animals

The animal care procedures were performed in accordance with the “Manual de Normas de Bioseguridad” (Comisión Nacional de Ciencia y Tecnología, CONICYT) and the Animal Care and Use Committee of the University of Concepcion (Concepción, Chile). Eighteen adolescent (1 month-old) male Pirbright guinea pigs (Instituto de Salud Pública, Santiago, Chile) were used in this study. The animals were housed under a 12-h light/dark cycle with food and water available ad libitum. No hierarchical fights were observed. Control animals (*n* = 7) were fed rabbit pellet and vegetables, whereas vitamin-C-deficient animals were not fed vegetables for 14 (*n* = 4) or 21 days (*n* = 7). All animals were kept under observation after scurvy induction according to the experimental animal care guidelines of the University of Concepcion to evaluate possible animal suffering before sacrifice. A loss of over 20% of the animal’s weight was considered to be the human endpoint of the study. The experimental protocols were approved by the Concepción University Licensing Committee, grant numbers 1181243 and 1140477.

### 2.2. Tissue Processing

After they were anesthetized with a mixture of ketamine (60 mg/kg), xylazine (10 mg/kg) and acepromazine maleate (10 mg/kg), guinea pigs were perfused transcardially with 0.9% saline and then with 4% paraformaldehyde (PFA) in 0.1 M phosphate buffer (PB) (*n* = 2) or Bouin’s fixative (*n* = 12) for light microscopy, or 2% PFA/2.5% glutaraldehyde in 0.1 M PB (*n* = 4) for electron microscopy. Brains were removed and post-fixed by immersing in the same fixative overnight at 4 °C. Bouin-fixed tissues were embedded in paraffin and cut into sequential 7 μm frontal sections. PFA-fixed tissues were cryo-protected by immersion in 30% sucrose for 24 h and then embedded in NEG50^TM^ (Thermo-Scientific, Waltham, MA, USA) to cut 50 μm frontal sections using a cryostat (MICROM HM520, Walldorf, Germany). PFA/glutaraldehyde-fixed tissues were cut into 150 μm frontal sections using a vibratome (Leica VT 1000S, Deer Park, IL, USA). For histological and immunohistochemical analysis, the tissues from 14 animals were analyzed, and tissues from four animals were analyzed for electron microscopy (Table S1).

### 2.3. Immunohistochemistry

After the 7 μm-thick sections were deparaffinized and rehydrated, endogenous peroxidase activity was inhibited by treatment with 3% H_2_O_2_ in methanol for 15 min. The sections were rinsed in Tris-HCl phosphate buffer (10 mM Tris, 120 mM NaCl, 8.4 mM Na_2_HPO_4_ and 3.5 mM KH_2_PO_4_; pH 7.8) and then incubated overnight at room temperature with the following primary antibodies diluted in 1% bovine serum albumin (BSA) in Tris-HCl phosphate buffer: anti-βIII-tubulin (1:1000, Promega, Madison, WI, USA), anti-PCNA (1:400, DAKO, Carpinteria, CA, USA) and anti-4-bromo-2′-deoxyuridine (BrdU; 1:200 Roche, Penzberg, Germany). The sections were rinsed and subsequently incubated for 2 h at room temperature with HRP-conjugated mouse anti-IgG (Jackson ImmunoResearch, West Grove, PA, USA). The peroxidase activity was developed using diaminobenzidine and H_2_O_2_. For immunohistochemical analysis of BrdU, the sections were treated with 2N HCl for 30 min at 37 °C and incubated with 5% BSA for 30 min before being incubated with the primary antibody. The images were obtained using an Axioplan 2 microscope (Carl Zeiss, Oberkochen, Germany) connected to a digital camera (Nikon, Digital Camera DXM1200, Melville, NY, USA). In all cases, omission of the primary antibody served as the negative control (Figure S1).

### 2.4. Multi-Labeling Immunofluorescence and Confocal Microscopy

Sections (50 μm-thick) were rinsed in Tris-HCl phosphate buffer and then co-incubated overnight at room temperature with lectin-diluted with 1% BSA in Tris-HCl phosphate buffer or with the following primary antibodies: anti-vimentin (1:200, Millipore, Darmstadt, Germany); anti-βIII-tubulin (1:1000, Promega) and FITC-conjugated isolectin B4 (1:10, Sigma, St. Louis, MO, USA). After the sections were rinsed, they were co-incubated for 2 h at room temperature with the following fluorophore-conjugated secondary antibodies: Cy5-conjugated chicken anti-IgG (1:200, Jackson ImmunoResearch, Baltimore Pike, West Grove, PA, USA) and DyLight 547-conjugated mouse anti-IgG (1:200, Jackson ImmunoResearch). The sections were also incubated in Hoechst 33258 (Sigma, St. Louis, MO, USA) as a nuclear stain. Multi-labeled images were obtained by confocal spectral microscopy (LSM 780, Carl Zeiss). Z-sectioning was performed at 1 μm intervals, and optical stacks of at least 30 images were used for the analysis. Digital three-dimensional (3D) reconstructions were created using Zeiss LSM software (ZEN, Carl Zeiss Microscopy GmbH, Aalen, Germany).

### 2.5. Transmission Electron Microscopy 

The 150 μm sections were washed in PB and immersed in 2% osmium tetroxide in PB for 1 h. After a washing step, samples were stained with 2% uranyl acetate in 70% ethanol for 3 h at 4 °C, dehydrated in ascending ethanol concentrations and incubated with propylene oxide for Araldite embedding. Then, sections were cured for 3 days at 60 °C. Serial semi-thin sections (1.5 μm) were cut on an ultramicrotome (Leica, Deer Park, IL, USA) and further stained with 1% toluidine blue. Finally, ultrathin (60 nm) sections were cut using a diamond knife on the ultramicrotome and examined under a Jeol Jem-1400 electron microscope (Jeol, Dearborn Road, Peabody, MA, USA). 

### 2.6. In Vivo BrdU Labeling

Control and vitamin-C-deficient guinea pigs received an intraperitoneal (i.p.) injection of BrdU (Sigma) (100 mg/kg body weight per injection). Four hours after the injection, animals were sacrificed for the proliferation analysis.

### 2.7. Cell Quantification and Statistical Analysis

For each marker of interest (anti-βIII-tubulin and anti-BrdU), five sections (every 210 μm) from each region (LVE and SVZ) from each animal were immunolabeled. Sections were chosen from the same area within each region. After images around the entire ventricular cavity were obtained using a 20× objective, the ventricle was reconstructed using Canvas X software (ACD Systems International Inc, Victoria, BC, Canada) and the total number of positive cells around the ventricular cavity in one hemisphere was quantified using Image J software (ImageJ 1.53a, National Institute of Health, Bethesda, MD, USA). Data represent the mean ± SD of each region for three animals. Statistical comparisons between two or more groups of data were carried out using analysis of variance (ANOVA) followed by Bonferroni post-tests. A *p*-value < 0.05 was considered to be statistically significant. GraphPad software (Prism 9, GraphPad Software, Inc., La Jolla, CA, USA) was used for all data analyses.

## 3. Results

### 3.1. Decreased Number of Neuroblasts and Proliferative Cells in SVZ and LVE with Vitamin C Deficiency in Adult Guinea Pigs

**Figure 1:**
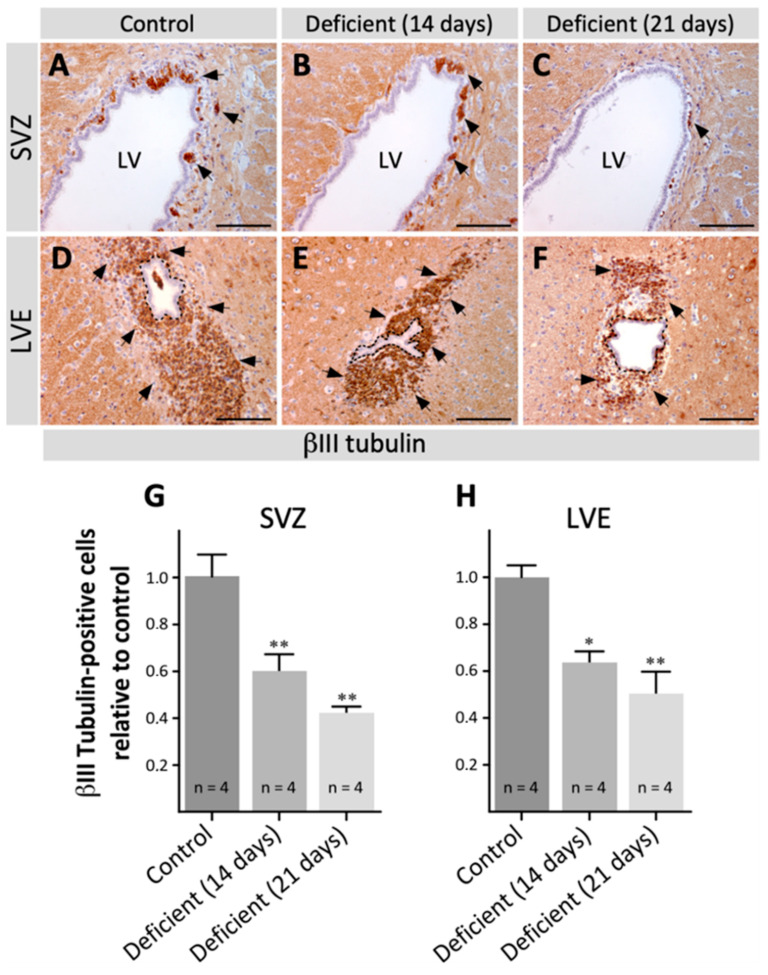
Analysis of neuroblasts in the brain of adult vitamin-C-deficient guinea pigs. Coronal sections of the brain of control guinea pigs (**A**,**D**) and the brain of guinea pigs with 14 (**B**,**E**) and 21 days (**C**,**F**) of vitamin C deficiency, labeled with anti-βIII tubulin (1:1000) to identify neuroblasts. In the SVZ (**A**–**C**) and LVE (**D**–**F**), neuroblasts progressively decreased with increasing days of vitamin C deficiency (arrows). (**G**,**H**) Neuroblasts were quantified in the SVZ (**G**) and LVE (**H**) of control and vitamin-C-deficient animals. The number of neuroblasts decreased progressively from the control animals to the 21-day deficient animals in both the SVZ and LVE. Data are presented as mean ± SD. Statistical analysis was performed using one-tailed ANOVA test and Bonferroni post-test; * *p* < 0.05, ** *p* < 0.01. *n* = 4. SVZ: subventricular zone. LV: lateral ventricle. LVE: lateral ventricle extension. Scale bar: 100 μm.

To determine if vitamin C deficiency alters the production of neuroblasts in the SVZ and its presence in the LVE, coronal sections of the SVZ and LVE of vitamin-C-deficient and control animals were immunolabeled with anti-βIII tubulin to detect neuroblasts. In the SVZ, neuroblasts were found in the subependymal area (Figure 1A–C, arrows) with progressively reduced numbers coinciding with the days of deficiency (Figure 1A–C, arrows). In the LVE, neuroblasts maintained their subependymal location; however, their amount gradually decreased as the days of vitamin C deficiency elapsed (Figure 1D–F, arrows). To confirm these changes, neuroblasts in the SVZ (Figure 1G) and in the LVE (Figure 1H) were quantified. Both in the SVZ and LVE, the decrease in neuroblasts was significant and progressive with the days of vitamin C deficiency. Moreover, the decrease reached 41.67 ± 0.05 % in the SVZ (Figure 1G) and 49.92 ± 0.19% in the LVE (Figure 1H) in animals with 21 days of vitamin C deficiency.

**Figure 2:**
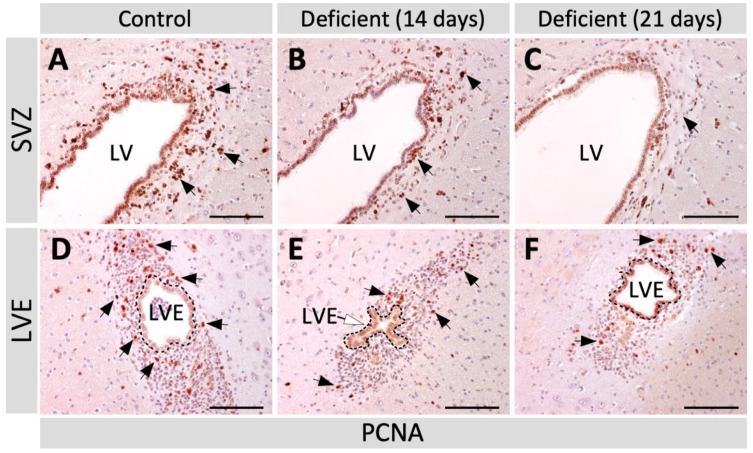
Analysis of PCNA-positive cells (B-type and C-type cells) in the brain of adult vitamin-C-deficient guinea pigs. Coronal sections of the brain of control guinea pigs (**A**,**D**) and the brain of guinea pigs with 14 (**B**,**E**) and 21 days (**C**,**F**) of vitamin C deficiency, labeled with anti-PCNA (1:100) as a proliferation marker. In the SVZ (**A**–**C**), PCNA-positive cells (arrows) progressively decreased with increasing days of vitamin C deficiency. In the LVE (**D**–**F**), PCNA-positive cells (arrows) decreased in vitamin-C-deficient animals (**E**,**F**). LVE: lateral ventricle extension. LV: lateral ventricle. SVZ: subventricular zone. *n* = 4. Scale bar: 100 μm.

**Figure 3:**
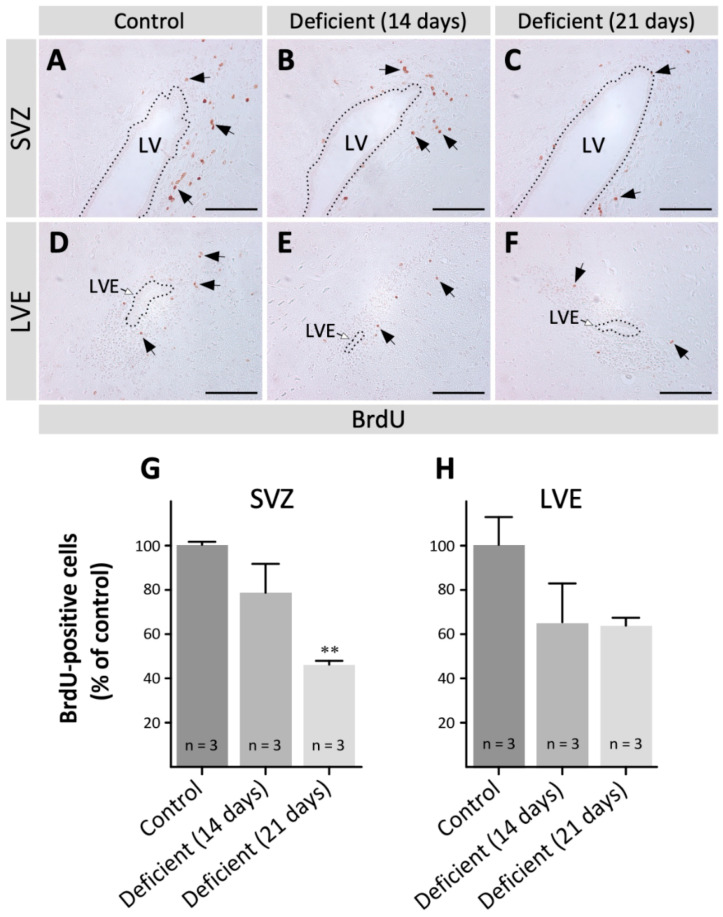
Analysis of BrdU-positive cells (B-type and C-type cells) in the brain of adult vitamin-C-deficient guinea pigs. Coronal sections of the brain of control guinea pigs (**A**,**D**) and the brain of guinea pigs with 14 (**B**,**E**) and 21 days (**C**,**F**) of vitamin C deficiency, labeled with anti-BrdU (1:200) as a proliferation marker. (**A**–**C**) In the SVZ, many BrdU-positive cells are observed in control animals (**A**) but they progressively decreased in guinea pigs with 14 (**B**) and 21 days (**C**) of vitamin C deficiency (arrows). (**D**–**F**) In the LVE, few BrdU-positive cells are observed in control animals (**D**) and even fewer in vitamin-C-deficient animals (**E**,**F**, arrows). (**G**,**H**) BrdU-positive cells were quantified in the SVZ (**G**) and LVE (**H**); their number decreased in the condition of vitamin C deficiency. Data are presented as mean ± SD. Statistical analysis was performed using one-tailed ANOVA test and Bonferroni post-test; ** *p* < 0.01. *n* = 3. LV: lateral ventricle. LVE: lateral ventricle extension. SVZ: subventricular zone. Scale bar: 100 μm.

To determine whether the decrease in neuroblasts found in deficient animals was associated with a reduction in the number of proliferative cells in the neurogenic regions, coronal sections of the SVZ and LVE of vitamin-C-deficient and control animals were immunolabeled with anti-PCNA to identify proliferative cells. In the SVZ, PCNA-positive cells were found in the subependymal area (Figure 2A, arrows) and their number decreased progressively (Figure 2A–C, arrows). Likewise, a decrease in PCNA-positive cells was also observed in the LVE of deficient animals (Figure 2D–F). To corroborate the results obtained through the anti-PCNA labeling, in vivo bromodeoxyuridine (BrdU) labeling was performed to specifically detect cells in the S-phase of the cell cycle. Then, coronal sections of the SVZ and LVE of vitamin-C-deficient and control animals were immunolabeled with an anti-BrdU antibody to detect proliferative cells and, subsequently, the number of BrdU-positive cells was quantified. In both the SVZ (Figure 3A–C) and LVE (Figure 3D–F), the number of BrdU-positive cells decreased in vitamin-C-deficient animals. In the SVZ, there was a significant decrease in BrdU-positive cells at 21 days of vitamin C deficiency (Figure 3G), while there was only a downward trend in the LVE (Figure 3H). Both PCNA and BrdU analysis demonstrated a decrease in the number of proliferative cells, suggesting a reduction in the proliferation of SVZ and LVE cells.

**Figure 4:**
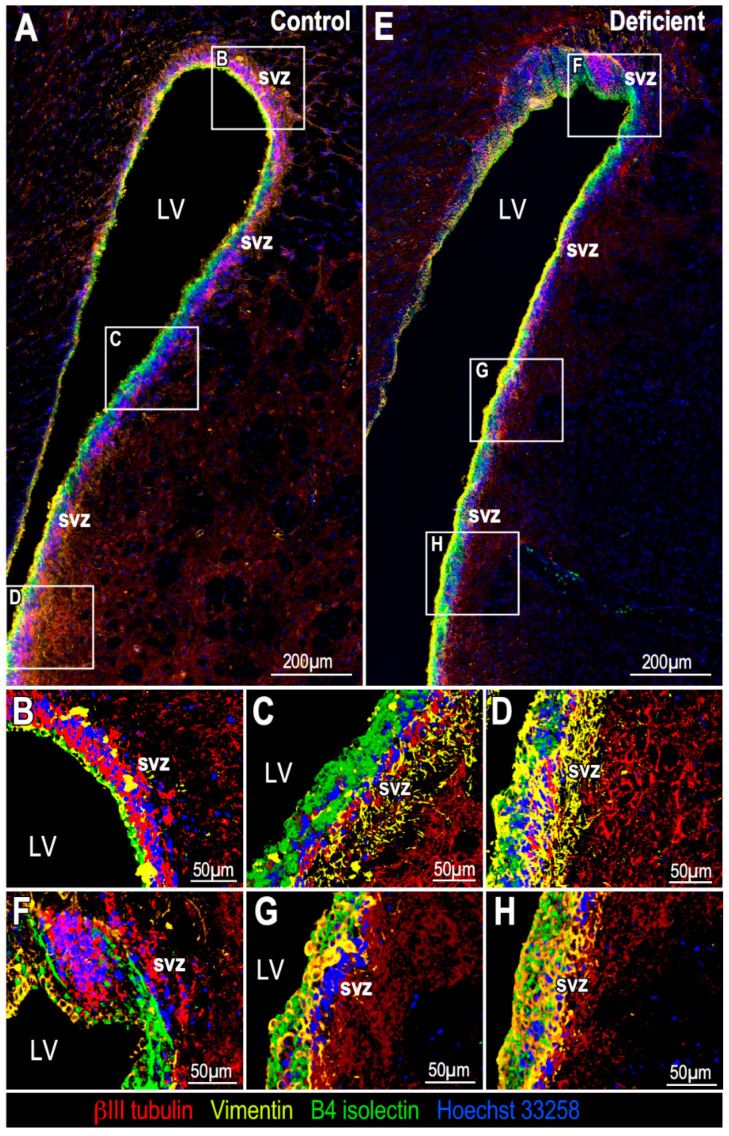
Multi-labeling analysis of neuronal and glial distribution in the SVZ of adult vitamin-C-deficient guinea pigs. Z-stacks projections, 3D reconstruction and tile scanning, using multiple markers: anti-βIII tubulin (1:1000, red), anti-vimentin (1:100, yellow), B4 isolectin (1:10, green) and Hoechst 33258 (1:1000, blue). (**A**) Glial and neuronal distribution in the SVZ of control guinea pigs. (**B**–**D**) Higher magnification of dorsal (**B**), medial (**C**) and ventral (**D**) areas of the LV in image A. (**E**) Glial and neuronal distribution in the SVZ of guinea pigs with 21 days of vitamin C deficiency. (**F**–**H**) Higher magnification of dorsal (**F**), medial (**G**) and ventral (**H**) area of the LV in image E; distribution of glial cells was different and the amount of neuroblasts (red) was lower along the LV of vitamin-C-deficient guinea pigs regarding control animals. LV: lateral ventricle. SVZ: subventricular zone.

To define whether vitamin C deficiency alters the cellular distribution of SVZ, coronal sections of the SVZ from control animals and from animals with 21 days of vitamin C deficiency were immunolabeled with anti-βIII tubulin to detect neuroblasts, anti-B4 isolectin to identify ependymal cells and anti-vimentin to identify ependymal cells and glial cells. Afterwards, 3D projections of Z-stacks of high-resolution confocal spectral images were generated (Figure 4). Although the neurogenic niche, and consequently, the clusters of neuroblasts, have always been associated with the most dorsal part of the LV, it is also possible to observe them in the medial and ventral areas of the LV (Figure 4A–D with higher magnification). In the vitamin-C-deficient animals, however, neuroblasts persisted in the dorsal part of the LV (Figure 4E,F) but practically disappeared in the medial (Figure 4E,G) and ventral (Figure 4E,H) parts of the LV, suggesting that the extent of neuroblast decrease could vary in different areas of the LV. This observation could apply not only in the dorso–ventral direction, but also in the rostro–caudal direction. For its part, the ependymal line was observed without changes between both conditions, but this was not so in glial cells, which seemed to have been redistributed. In control animals, glial cells seemed to cover a larger space in the SVZ (Figure 4C,D); however, in deficient animals, they seemed to be mostly distributed in the area closer to the ventricle cavity (Figure 4G,H), an area where neuroblasts are usually found in control conditions. 

**Figure 5:**
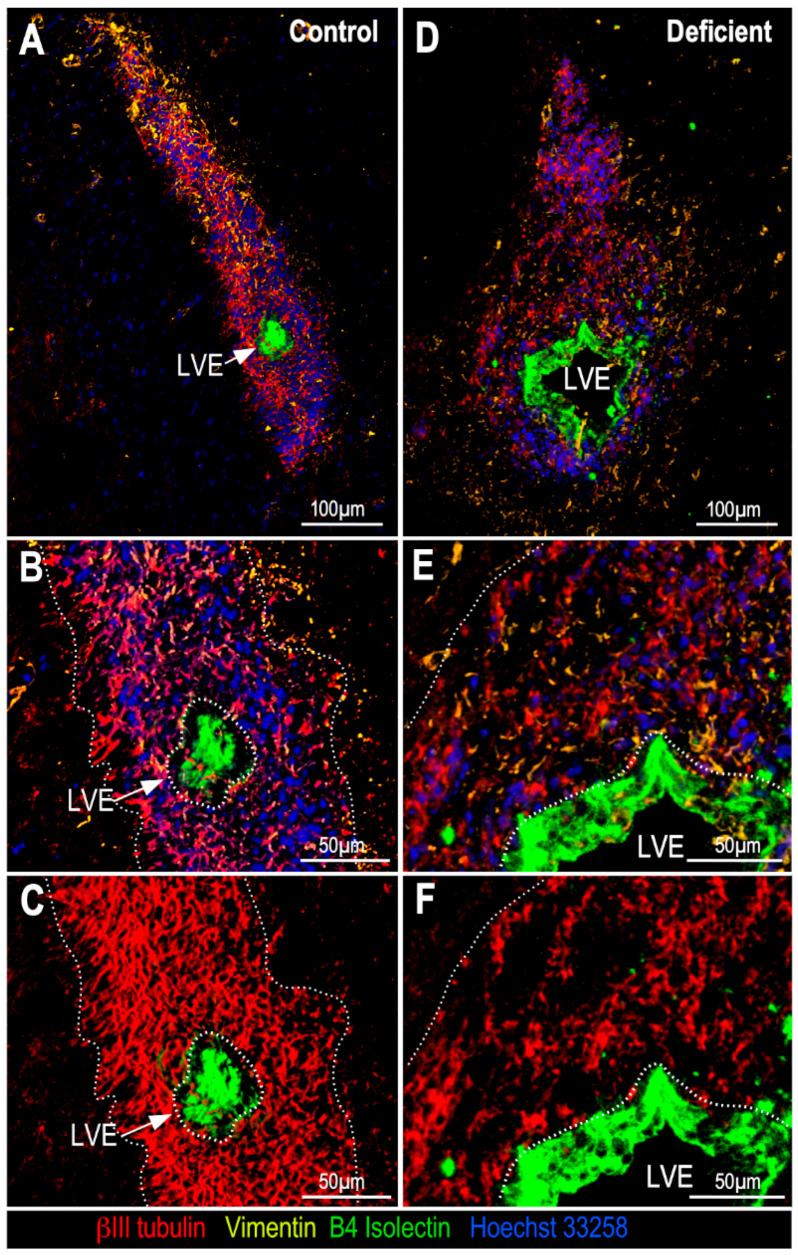
Multi-labeling analysis of neuronal and glial distribution in the LVE of adult vitamin-C-deficient guinea pigs. Z-stacks projections, 3D reconstruction and tile scanning, using multiple markers: anti-βIII tubulin (1:1000, red), anti-vimentin (1:100, yellow), B4 isolectin (1:10, green) and Hoechst 33258 (1:1000, blue). (**A**) Distribution of glial and neuronal cells in the LVE of control guinea pig brain. (**B**) Higher magnification of the LVE in image A. (**C**) Same image as in panel B, without blue (nuclei) and yellow (glial cells) channels. (**D**) Glial and neuronal distribution in the LVE of the brain of guinea pigs with 21 days of vitamin C deficiency. (**E**) Higher magnification of the LVE in image D. (**F**) Same image as in panel E, without blue (nuclei) and yellow (glial cells) channels. Here, the density of neuroblasts (red) was much lower than in the control (**C**, dotted lines); however, the number of glial cells (yellow) was similar. LVE: lateral ventricle extension.

Analysis of the LVE showed that apart from the evident decrease in the density of neuroblasts surrounding the LVE, an increase in the number of glial cells was observed in deficient animals (Figure 5E,F). Regarding the size of the LVE (Figure 5A,D), it is important to note that its diameter can vary notoriously from one animal to another [23].

**Figure 6:**
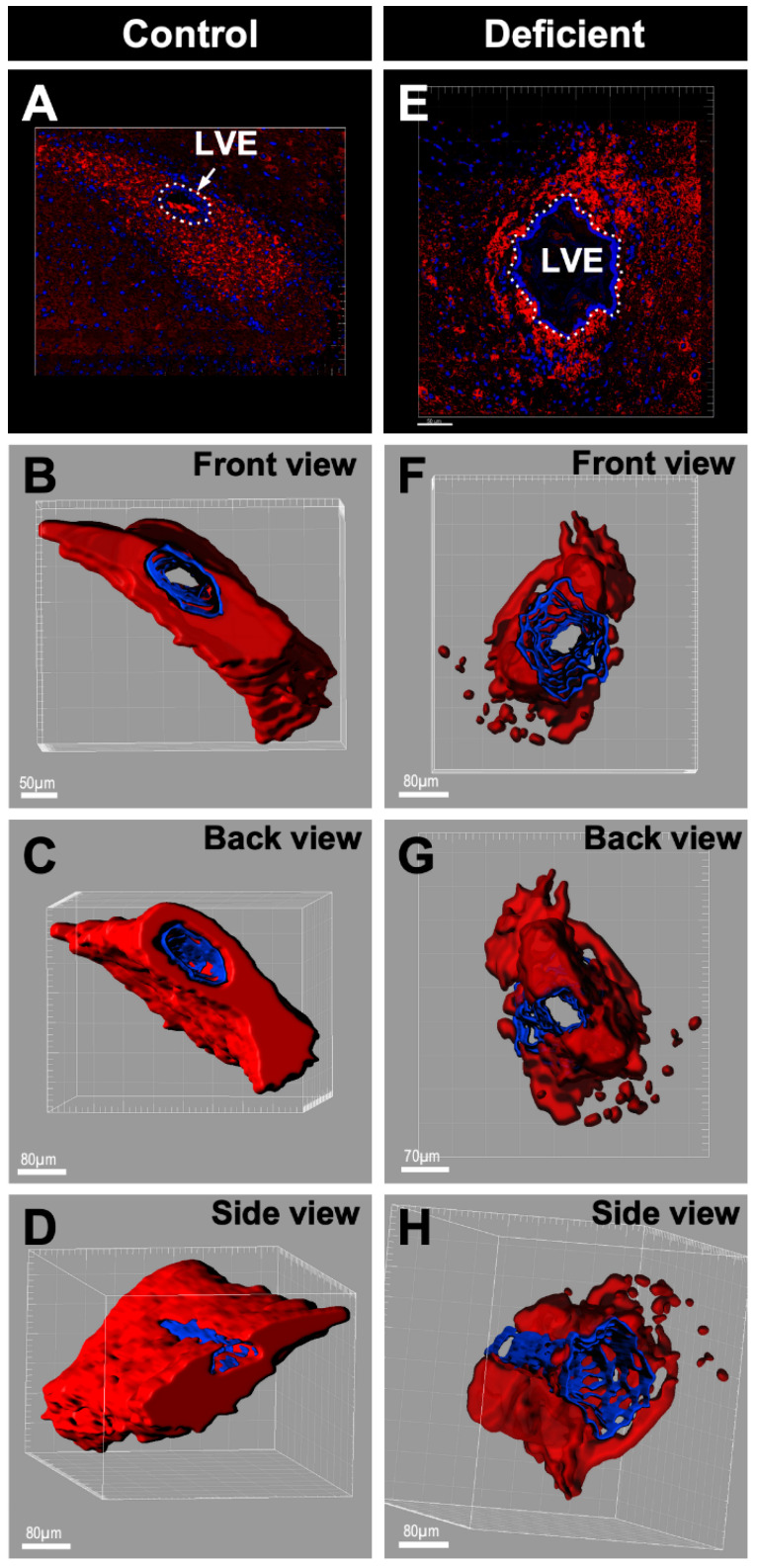
Three-dimensional reconstruction of the LVE and surrounding neuroblasts of control and vitamin-C-deficient guinea pigs. 3D reconstruction of Z-stacked coronal sections of the LVE of control guinea pigs and guinea pigs with 21 days of vitamin C deficiency labeled with anti-βIII tubulin (red, 1:1000) and hematoxylin (blue). (**A**) Z-stacked coronal sections of the LVE of control guinea pig. (**B**–**D**) Three different rotations of the 3D-reconstruction of the Z-stacked coronal sections in image A. (**E**) Z-stacked coronal sections of the LVE of guinea pigs with 21 days of vitamin C deficiency. (**F**–**H**) Three different rotations of the 3D-reconstruction of the Z-stacked coronal sections in image E. LVE: Lateral ventricle extension. Scale bar for images (**A**) and (**E**): 50 μm.

To demonstrate that the decrease in neuroblasts is not restricted to a particular rostro–caudal area and can be observed throughout the entire LVE, a 3D reconstruction of 560 μm throughout the LVE was performed. One out of every five sections (serial sections) of the brain of vitamin-C-deficient and control animals were immunolabeled with anti-βIII-tubulin; then, the images obtained were stacked using IMARIS^®^ (Figure 6). The results confirmed that the decrease in neuroblasts occurred throughout the entire LVE of the vitamin-C-deficient guinea pig (Figure 6F–H) relative to the control guinea pig (Figure 6B–D).

### 3.2. Vitamin C Deficiency Alters Cell Morphology and Cell Composition of the SVZ and LVE in Adult Guinea Pig Brain

**Figure 7:**
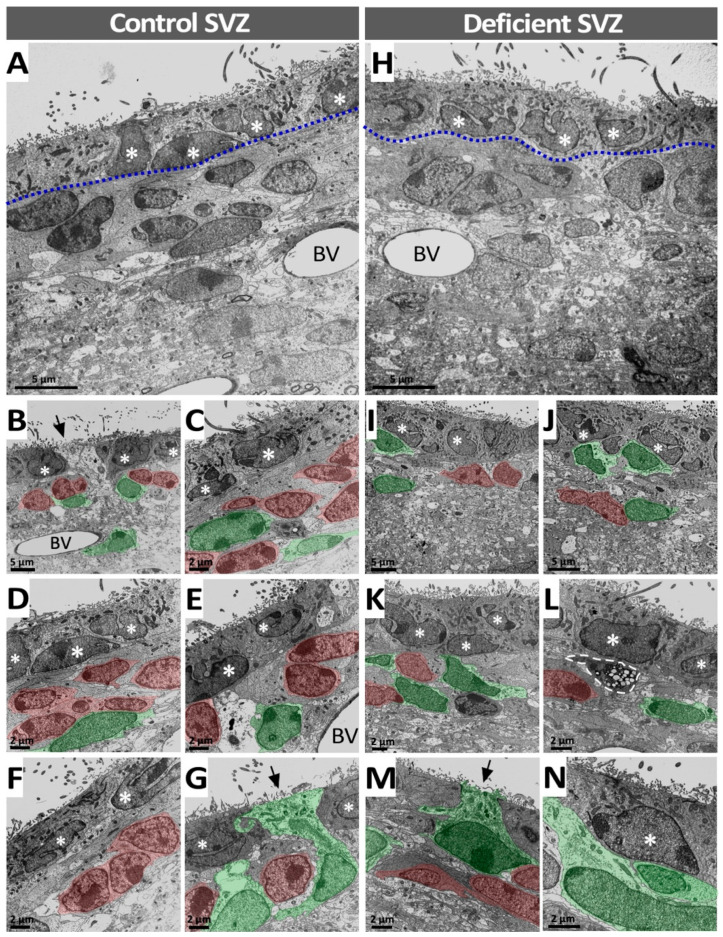
Ultrastructural analysis of the SVZ in vitamin-C-deficient guinea pigs. (**A**–**G**) Ultrathin sections of the SVZ in control guinea pigs; the ciliated ependymal cell line (dotted line) and different cell types in the subependymal area are observed (**A**). The ependymal cells (white asterisks) show cilia and apical microvilli, an irregular nucleus and an electron-dense cytoplasm (**B**–**G**). In the subependymal zone, neuroblasts (in red) have a heterochromatic nucleus and a scanty, electron-dense cytoplasm (**B**–**G**). Astrocytes (in green) have a euchromatic nucleus, an electron-lucent cytoplasm and often contact the ventricle (**G**, arrow). C-type cells are not detected in this set of images. (**H**–**N**) Ultrathin sections of the SVZ of vitamin-C-deficient guinea pigs showed that the cell distribution was similar to control SVZ; however, the number of cells was altered. A decline in the number of neuroblasts was observed and, consequently, cells in the subependymal area were mainly astrocytes (**I**–**N**). In addition, fewer astrocytes were observed contacting the ventricle (**M**, arrow). Apoptotic cell (dashed line) (**L**). LV: lateral ventricle. BV: blood vessel.

To determine if vitamin C deficiency induces changes in the cytoarchitecture of the SVZ and LVE or in the morphology of their cells, their ultrastructure was explored through transmission electron microscopy, and SVZ and LVE cell types were identified according to their ultrastructural features [23]. In the SVZ, ependymal cells were identified by cilia and microvilli on their apical surface by their irregular nucleus and their electron-dense cytoplasm (Figure 7A–G, asterisks); neuroblasts were identified by their sparse electron-dense cytoplasm and their heterochromatic nucleus (Figure 7B–G, cells in red). Astrocytes were identified by their electron-lucid cytoplasm and euchromatic nucleus (Figure 7B–G, cells in green), and type C cells by their electron-lucid cytoplasm and their irregular and large nucleus with numerous small clusters of condensed chromatins. However, it is worth mentioning that no type C cells were found in the images selected for the figure. 

When the SVZ ultrastructure of control and vitamin-C-deficient animals were compared, no significant changes in cell morphology were observed. In the SVZ of deficient animals (Figure 7H–N), the ependymal cells maintained their apical cilia and microvilli, and their nuclei (asterisks) were observed as irregular as in the SVZ of control animals. Furthermore, neuroblasts (Figure 7H–N, cells in red) and astrocytes (Figure 7H–N, cells in green) also retained their distinctive ultrastructural features. Regarding the cellular composition of the SVZ in deficient animals, the number of cells forming part of the cellular architecture was notoriously lower when compared with the control (Figure 7H–N). Although the number of astrocytes and ependymal cells seemed to remain unchanged, there was a marked decline in the number of neuroblasts.

**Figure 8:**
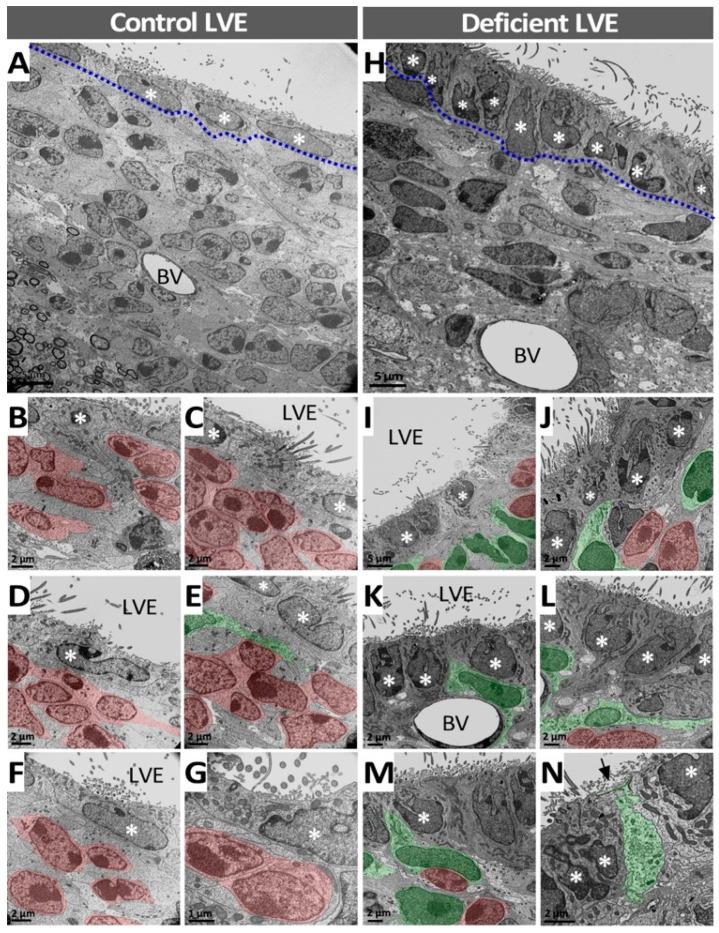
Ultrastructural analysis of the LVE of vitamin-C-deficient guinea pigs. (**A**–**G**) Ultrathin sections of the LVE of control guinea pigs; the ciliated ependymal cell line (dashed line) and a large number of cells in the subependymal area is identified (**A**). Neuroblasts (in red) and astrocytes (in green) exhibit a similar morphology to that described in the control SVZ; however, the ependymal cell nucleus (white asterisks) is more regular in the LVE (**B**–**G**) compared with the control SVZ. Most of the subependymal cells correspond to neuroblasts (**B**–**G**). (**H**–**N**) Ultrathin sections of the LVE of vitamin-C-deficient guinea pigs show that the cell distribution was similar to control LVE. However, the number of cells was dramatically decreased and the morphology of the ependymal cell nuclei (white asterisks) was significantly altered (**H**–**N**). Neuroblasts were reduced in number (cells in red), while the number of astrocytes (cells in green) increased (**I**–**N**). BV: blood vessel. LVE: lateral ventricle extension.

Unlike that observed in the SVZ, differences between control and vitamin-C-deficient animals were found in the cell morphology of the LVE. In control animals, the ependymal cells had a flat shape and a regular nucleus (Figure 8A–G, asterisks), while the LVE of deficient animals had a cubic shape and an irregular nucleus (Figure 8H–N, asterisks). In the subependymal area of the LVE, the morphology of the cells was also different; nuclei with a more regular size and shape were observed in the control LVE (Figure 8A–G) compared with the LVE of a deficient animal (Figure 8H–N). The cytoarchitecture of the LVE of deficient animals also showed important differences compared with the control. Specifically, a large decrease in the number of neuroblasts was observed in the LVE of deficient animals (Figure 8H–N, cells in red), while astrocytes seemed to increase their number (Figure 8H–N, cells in green). Consequently, variation in the number of cells resulted in changes to their location. In the control LVE, neuroblasts were situated below the line of ependymal cell (Figure 8A–G, cells in red); however, in deficient animals, astrocytes were observed in that location (Figure 8H–N, cells in green).

## 4. Discussion

In the present work, we described the effect of vitamin C deficiency on neurogenesis, and the cellular composition and morphology of the SVZ and LVE from adult guinea pig brain. We found that neuroblasts and proliferating cells in the SVZ and LVE are progressively and significantly reduced in proportion with the number of days under vitamin C deficiency. This reduction is not restricted to a particular area and is observed throughout the LVE. We also found that vitamin C deficiency alters the cellular morphology at the ultrastructural level, especially in cells from the LVE.

Vitamin C deficiency in adult guinea pig induced a progressive reduction in neuroblasts in the SVZ and LVE. This reduction was higher in 50% of the animals consuming a deficient diet for 21 days, which is consistent with a previous study from our group in which we showed that neurospheres isolated from the SVZ of adult rat brain treated with 200 μM vitamin C induced the production of new neurons through differentiation towards a neuronal lineage [9]. Thus, we hypothesized that reducing vitamin C produces the opposite effect (i.e., a decline in the production of neuroblasts). In addition, another study showed a significant reduction in the number of neurons in three different regions of the hippocampus from animals under vitamin C deficiency [13]. Overall, these findings show a vital role of vitamin C in adult brain neurogenesis. 

We also showed that vitamin C deficiency progressively reduced the number of proliferating cells in SVZ and LVE, suggesting that the decrease in neuroblasts is directly associated with the lower proliferation. A previous study described that prenatal vitamin C deficiency induced a significant reduction in the volume of the postnatal hippocampus, attributed to reduced migration of neuroblasts towards the granular layer of the dentate gyrus [26], without considering a decrease in the proliferation of precursor cells nor reduced survival rate of new neurons [26]. The discrepancies between this [26] and the present study may be due to the inherent differences of the neurogenic niches. For instance, the proliferative capacity of the hippocampal dentate gyrus is lower at the SVZ [27,28], making this zone more likely to be affected. Furthermore, in the rat, BrdU-positive proliferative cells at the SVZ and RMS express the vitamin C transporter, SVCT2 [9], suggesting a role for vitamin C in the regulation of proliferative cells in the SVZ and LVE.

In the SVZ, NSCs proliferate to give rise to transient precursors, which in turn, and depending on the environment signals, can differentiate into cells of glial lineage, such as astrocytes and oligodendrocytes, or neuronal lineage, such as neuroblasts [27,28]. Based on this, the reduction in neuroblasts induced by vitamin C deficiency may be explained by (i) apoptosis of NSCs, transient precursors and/or neuroblasts; (ii) increased differentiation into the glial lineage in detriment of the neuronal lineage; and/or (iii) reduction in the proliferation of NSCs and/or transient precursors. First, we were unable to evaluate apoptosis because Bouin’s fixative is not compatible with the TUNEL assay and immunostaining of cleaved caspase-3 did not work as expected. Nevertheless, we paid special attention to the presence of cells with apoptotic morphology during the ultrastructural analysis, finding no differences between control and deficient animals. Second, even when we observed a subtle increase in the number of glial cells in the EVL, it is unlikely that this increase could be due to an increase in glial cells generation, either by proliferation or differentiation from NSCs, since NSCs have not been detected in this area [23]. It is more likely that the increase was related to a redistribution of cells, triggered by the absence of neuroblasts that occupy the areas closest to the ventricular cavity. Thus, we propose that vitamin C deficiency reduces the number of neuroblasts via lower proliferation of NSCs and/or transient precursors, therefore impacting the genesis of neuroblasts. This hypothesis fits our findings of reduced proliferation and number of neuroblasts, without any significant variation in the number of glial cells, which is further supported by previous studies describing the positive effect of vitamin C on the proliferation and differentiation of NSCs, precursors and neuroblasts [29,30,31]. 

Many mechanisms could account for the effect of vitamin C deficiency on the reduced proliferation of NSCs and/or precursor cells: (i) a direct action over the expression of proteins regulating proliferation and differentiation of NSCs, (ii) an indirect action due to increased oxidative stress and/or (iii) an indirect action due to reduced collagen synthesis. Regarding the first possibility, vitamin C is known to affect the epigenetic landscape of human embryonic stem cells through inducing specific histone demethylation events and the expression of certain genes [32]. This function could be related with the capacity of vitamin C to modulate the activity of Fe(II)/2-oxoglutarate-dependent oxygenases [33]—among them, histone demethylases Jhdm1a/1b [34] and DNA demethylase TET1 [35,36,37]. Regarding oxidative stress, it is widely documented that vitamin C deficiency increases reactive oxygen species (ROS) [38,39,40,41], which has a negative effect on NSC proliferation through inhibition of ERK1/2 [42]. Moreover, embryos from SOD2-null mice, which exhibit high levels of superoxide in the brain, are characterized by reduced neurogenesis in the SVZ, attributed to a reduced proliferative capacity of NSCs [42]. Lastly, in relation to collagen modulation, it is known that the basal lamina of blood vessels in the SVZ is mainly composed of collagen and laminin and is projected towards the subependymal zone [43], where collagen and laminin integrate diverse factors modulating neurogenesis through the astrocytic foot processes [44] (Mercier y col., 2002). Therefore, vitamin C deficiency could affect synthesis and deposition of collagen, reducing neurogenesis.

At the ultrastructural level, we observed that vitamin C deficiency is associated with a reduction in neuroblasts and a subtle increase in astrocytes in the SVZ and LVE, whereas ependymal cells remain unaffected. These changes also impact the distribution of these cells in a way that neuroblasts are localized immediately under the ependymal line in the LVE from control animals, while astrocytes occupy this place in LVE from deficient animals. Moreover, we observed relevant morphological differences in the LVE, but not in the SVZ, from deficient animals compared with the controls. The ependymal cells are flatter and show regular nuclei in control animals, whereas they show a cuboidal shape with irregular nuclei in deficient animals. Ependymal cells also show pleomorphic nuclei in the LVE from deficient animals. Evidence suggests that vitamin C deficiency induces nuclear and cellular morphological changes through increased oxidative stress and reorganization of the actin cytoskeleton [45]. In addition, oxidative stress promotes telomere shortening and chromosomic instability, in turn, altering the nuclear morphology [46]. Nevertheless, the fact that the morphological alterations in deficient animals were more evident in the LVE may be due to a lower availability of vitamin C from the CSF, contributing to higher oxidative damage.

## 5. Conclusions

Our data show the relevance of vitamin C in proliferation, differentiation and neurogenesis in the SVZ from adolescent guinea pig brain and how these processes impact on the maintenance of the normal cytoarchitecture of the SVZ and LVE. Vitamin C deficiency remains a serious health problem for a high percentage of the world population. For instance, in the United Kingdom, 46% of men and 35% of women within the low-income population had vitamin C deficiency [47]. In the U.S., the prevalence of vitamin C deficiency, adjusted by age, is ~7% of the population, which is reduced with a higher socioeconomic status [2]. In the Canadian youth between 20 and 29 years old, 14% have vitamin C deficiency [3]. Considering that these data come from developed countries, we infer that the prevalence of vitamin C deficiency could be significantly higher in developing countries. Based on our results observed in guinea pig brain and given the well-known similarities between guinea pig and human brains, we propose that vitamin C deficiency could have important negative consequences in human neurogenesis, especially for children and pregnant women.

## Data Availability

The data presented in this study are available in the article and Supplementary Materials.

## Supplementary Materials

The following are available online at https://www.mdpi.com/article/10.3390/antiox11102030/s1, Figure S1: Negative controls without primary antibody in the SVZ of guinea pig brain, Table S1: Total number of animals used in the experiments.
