# Supplementary material for: Vitamin C Deficiency Reduces Neurogenesis and Proliferation in the SVZ and Lateral Ventricle Extensions of the Young Guinea Pig Brain
Source: Antioxidants (Basel). 2022 Oct 14;11(10):2030. doi: 10.3390/antiox11102030 (PMC9598632; doi:10.3390/antiox11102030)
Supplement: Supplementary file 1 [file antioxidants-11-02030-s001.zip › antioxidants-1937028-supplementary.pdf]

Supporting Online Material for

Vitamin C deficiency reduces neurogenesis and proliferation in the SVZ and lateral ventricle extensions of the young guinea pig brain

Nery Jara, Manuel Cifuentes, Fernando Martínez1, Iván González-Chavarría, Katterine Salazar, Lucas Ferrada and Francisco Nualart*.

*To whom correspondence should be addressed. E-mail: frnualart@udec.cl

Food composition information

Laboratory Rabbit Diet 5321

DESCRIPTION

Laboratory Rabbit Diet is a complete life-cycle rabbit diet formulated to support maintenance of research animals during reproduction, lactation, growth, and maintenance. This is a complete life-cycle pelleted ration formulated using managed formulation, delivering Constant Nutrition®. This is paired with the selection of highest quality ingredients to assure minimal inherent biological variation in long-term studies.

Features and Benefits

- Managed Formulation delivers Constant Nutrition®
- Versatile all-in-one life-cycle product
- Designed to support the energy requirements for reproduction, lactation, growth and maintenance

Product Forms Available	*Catalog #*
• Pellet, 5/32" x 3/8", 50 lb	0001366
Other Versions Available	***Catalog #***
• 5LS4: PicoLab® Laboratory Rabbit Diet, 30 lb	**3006744-220

** For ordering, contact info@LabDiet.com

GUARANTEED ANALYSIS

Crude protein not less than	16.00%
Crude fat not less than	2.50%
Crude fiber not less than	14.00%
Crude fiber not more than	18.00%
Moisture not more than	12.00%
Ash not more than	8.00%
Calcium not less than	0.70%
Calcium not more than	1.20%
Phosphorus not less than	0.50%
Salt not less than	0.25%
Salt not more than	0.75%
Sodium not more than	0.55%
Vitamin A not less than	9000 IU/lb
Vitamin E not less than	10 IU/lb

INGREDIENTS

Dehydrated Alfalfa Meal, Ground Corn, Dehulled Soybean Meal, Ground Soybean Hulls, Wheat Middlings, Ground Oats, Cane Molasses, Dicalcium Phosphate, Salt, Calcium Carbonate, Soybean Oil, DL-Methionine, Choline Chloride, Folic Acid, Vitamin A Acetate, Vitamin D3 Supplement, Magnesium Oxide, Pyridoxine Hydrochloride, Calcium Pantothenate, Vitamin E Supplement, Nicotinic Acid, Vitamin B-12 Supplement, Riboflavin Supplement, Manganous Oxide, Zinc Oxide, Ferrous Carbonate, Copper Sulfate, Zinc Sulfate, Calcium Iodate, Cobalt Carbonate, Sodium Selenite.

FEEDING DIRECTIONS

Laboratory Rabbit Diet should be self-fed except when weight control is necessary. Young rabbits will begin to consume feed when they come out of the nest box at approximately three weeks of age. Mature adult rabbits will consume approximately 4 to 6 oz. per day. Plenty of clean, fresh water should be available to the animals at all times.

For information regarding shelf life please visit www.labdiet.com.

CHEMICAL COMPOSITION[1]

Nutrients[2]

**Protein, %**	**17.5**
Arginine, %	0.97
Cystine, %	0.29
Glycine, %	0.76
Histidine, %	0.44
Isoleucine, %	0.88
Leucine, %	1.32
Lysine, %	0.91
Methionine, %	0.35
Phenylalanine, %	0.81
Tyrosine, %	0.52
Threonine, %	0.66
Tryptophan, %	0.20
Valine, %	0.82
Serine, %	0.84
Aspartic Acid, %	1.92
Glutamic Acid, %	3.13
Alanine, %	0.92
Proline, %	1.09
Taurine, %	0.00
**Fat (ether extract), %**	**2.8**
**Fat (acid hydrolysis), %**	**4.0**
Cholesterol, ppm	0
Linoleic Acid, %	1.08
Linolenic Acid, %	0.23
Arachidonic Acid, %	0.00
Omega-3 Fatty Acids, %	0.33
Total Saturated Fatty Acids, %	0.40
Total Monounsaturated Fatty Acids, %	0.48
**Fiber (Crude), %**	**14.9**
Neutral Detergent Fiber[3], %	30.3
Acid Detergent Fiber[4], %	20.0
**Nitrogen-Free Extract (by difference), %**	**48.2**
Starch, %	18.2
Sucrose, %	2.18
**Total Digestible Nutrients,%**	**65.9**
**Gross Energy, kcal/gm**	**3.39**
**Physiological Fuel Value[5], kcal/gm**	**2.88**
**Metabolizable Energy, kcal/gm**	**2.32**

Minerals

**Ash, %**	**6.2**
Calcium, %	0.95
Phosphorus, %	0.50
Phosphorus (non-phytate), %	0.31
Potassium, %	1.40
Magnesium, %	0.25
Sulfur, %	0.23
Sodium, %	0.30
Chloride, %	0.66
Fluorine, ppm	15
Iron, ppm	340
Zinc, ppm	110
Manganese, ppm	120
Copper, ppm	17
Cobalt, ppm	1.4
Iodine, ppm	1.6
Chromium (added), ppm	0.01
Selenium, ppm	0.55

Vitamins

Carotene, ppm	15
Vitamin K, ppm	3.0
Thiamin, ppm	4.6
Riboflavin, ppm	5.6
Niacin, ppm	50
Pantothenic Acid, ppm	19
Choline, ppm	1370
Folic Acid, ppm	8.4
Pyridoxine, ppm	4.5
Biotin, ppm	0.30
B_{12}, mcg/kg	7.0
Vitamin A, IU/gm	20
Vitamin D_3 (added), IU/gm	1.1
Vitamin E, IU/kg	45
Ascorbic Acid, mg/gm	0.0

Calories provided by:

Protein, %	23.307
Fat (ether extract), %	8.748
Carbohydrates, %	66.945

1. Formulation based on calculated values from the latest ingredient analysis information. Since nutrient composition of natural ingredients varies and some nutrient loss will occur due to manufacturing processes, analysis will differ accordingly.
2. Nutrients expressed as percent of ration except where otherwise indicated. Moisture content is assumed to be 10.0% for the purpose of calculations.
3. NDF = approximately cellulose, hemi-cellulose and lignin.
4. ADF = approximately cellulose and lignin.
5. Physiological Fuel Value (kcal/gm) = Sum of decimal fractions of protein, fat and carbo- hydrate (use Nitrogen Free Extract) x 4,9,4 kcal/gm respectively.

NOTE: When assayed, actual levels may vary from calculated values.

01/04/22 RHI-W 8

LabDiet®
www.labdiet.com

Table S1. Total number of animals used in the experiments.

Groups	Tissue processing	Microscopy
(4) control guinea pigs<br>(4) 14-day deficient guinea pigs<br>(4) 21-days deficient guinea pigs	Fixation with Bouin's fixative<br>7-µm paraffin slices	Bright-field microscopy
(1) control guinea pig<br>(1) 21-days deficient guinea pig	Fixation with 4% PFA<br>50-µm cryostat brain slices	Confocal fluorescence microscopy
(2) control guinea pigs<br>(2) 21-days deficient guinea pigs	Fixation with 2% PFA/2.5% glutaraldehyde<br>60-nm brain slices	Transmission electron microscopy

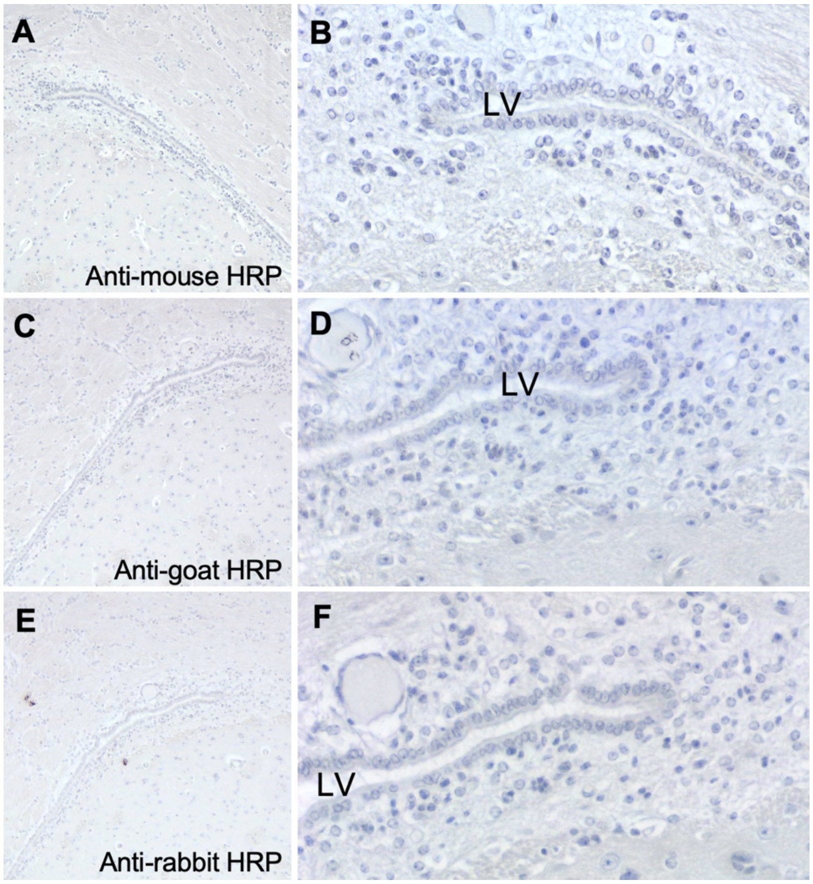

Figure S1. Negative controls without primary antibody in the SVZ of guinea pig brain. (A) anti-mouse HRP (1:200) labeled lateral ventricle and (B) same area at higher magnification. (C) anti-goat HRP (1:200) labeled lateral ventricle and (D) same area at higher magnification. (E) anti-rabbit HRP (1:200) labeled lateral ventricle and (F) same area at higher magnification. LV: lateral ventricle. Magnification for A, C, E is 100×; Magnification for B, D, F is 300×.
